# Influence of contextual factors on physical demands and technical-tactical actions regarding playing position in professional soccer players

**DOI:** 10.1186/s13102-021-00386-x

**Published:** 2021-12-16

**Authors:** Adrián Díez, Demetrio Lozano, Jose Luis Arjol-Serrano, Elena Mainer-Pardos, Daniel Castillo, Marcelino Torrontegui-Duarte, Hadi Nobari, Diego Jaén-Carrillo, Miguel Lampre

**Affiliations:** 1 Universidad San Jorge, Autov A23 km 299, 50830 Villanueva de Gállego, Zaragoza Spain; 2 Faculty of Health Sciences, Universidad Isabel I, Burgos, Spain; 3 Department Nursing and Podiatry, University of Malaga, Malaga, Spain; 4 Department of Exercise Physiology, Faculty of Educational Sciences and Psychology, University of Mohaghegh Ardabili, Ardabil, 56199-11367 Iran; 5 Department of Physiology, School of Sport Sciences, University of Extremadura, 10003 Cáceres, Spain

**Keywords:** Soccer, Performance, External training load, Contextual game factors, GPS

## Abstract

**Background:**

The aim of this study was to analyse the physical demands and technical-tactical actions for each playing position according to game location and final outcome in professional soccer players.

**Methods:**

A convenience sample was obtained from twenty-one professional male soccer players, belonged to same soccer team of the Spanish Second Division. Players’ physical demands were monitored during each match using a portable 18 Hz GPS unit and 600 Hz triaxial accelerometer. These analysed demands were total distance, moderate speed running distance (>14.4 km·h^-1^), high-speed running distance (>19.8. km·h-1), sprint distance (>25.0 km·h^-1^), number of accelerations between 2 and 4 m·s^-2^ and above 4 m·s^-2^, and number of decelerations between 2 and 4 m·s^-2^ and above 4 m·s^-2^. The data related to technical-tactical actions were obtained from WyScout®, a computerized multiple-camera tracking system based on the OPTA® track analysis tool. The obtained indicators were general, defensive and offensive.

**Results:**

For all players, higher total distance (p = 0.045; effect size [ES] = 0.24, small effect) was covered and greater deceleration 2-4 m·ss^-2^ (p = 0.001; ES = 0.68, medium effect) was performed when the team plays at home and lose and for all players, playing at home and winning demanded higher defensive volume (p =0.014; ES = − 1.49, large effect) and nº interceptions (p =0.031; ES = − 1.40, large effect) in comparison to playing at home and losing.

**Conclusions:**

The physical demands and technical-tactical actions vary when contextual game factors (i.e., match location and final outcome) are considered. We can confirm that, although the training of physical demands does not influence the final result of the match, the training of technical tactical actions could help to achieve an optimal performance of the team to win matches.

## Background

On the one hand, success in soccer is based on the ability of scoring a higher number of goals than the other team over a game [1, 2]. On the other hand, the first goal in a game [3–5] and a reduced number of goals conceded, especially during the second half of a season, have been associated with success in soccer [5]. Coaches and researchers have attempted to identify strategies to improve effectivity in both the opponent box (i.e., to score goals) and their own box (i.e., to avoid conceding goals) [6] by employing key performance indicators (KPIs) [7–10]. KPIs facilitate the objective analysis of performance over a game and are referred as the variables that define physical and technical-tactical performance aspects which contribute to success [11]. In addition, it would be interesting to identify KPIs in professional soccer so increase the likelihood of winning a game and to optimise soccer training and competition [1], understanding optimization as the proper adjustment of workloads.

The existing connection between physical efforts carried out in a game and their relation with success have been widely examined in the main European soccer leagues [12–15] and World Cup championships [16, 17]. Seemingly, physical demands are a poor indicator to determine team success over a season or championship. Regarding this, previous works concluded that the teams in the top 5 of Italian Serie A and English Premier League covered less total distance at a lower intensity than the rest of the teams [13, 14]. Similar findings were found in German Bundesliga and Spanish LaLiga where there is no correlation between final ranking position, total distance covered and intensity of efforts [12, 15]. However, to determine success in teams, it would be necessary a holistic perspective might be needed including technical-tactical skills as they are considered essential within soccer performance [13].

In professional soccer, accuracy when attempting the opponent goal followed by number of attempts, ball possession percentage time, and pass accuracy are identified as key actions when analysing performance [18]. It has been identified that in successful team’s possession time is influenced by type of start- up, intention and field zone. While, possession time of unsuccessful teams is determined fundamentally by intention and match status [19]. Greater overall ball possession near the opponent box has been identified as a good indicator of success since there are greater possibilities to score and thus to win [9].

Either facing the game as home or away team may also influence the final score. It has been found that local teams covered longer distances at low intensity (<14.1 km.h^-1^) what may suggest that this condition could jeopardise team performance [20]. In a deeper analysis it was suggested that the identification of game actions which predict soccer performance should be contextualised in terms of the role the team plays (i.e., home vs. away) since perhaps the local team might have increased their possibilities to win [7]. On the other hand, game location seems to have no influence on speed zones physical performance [21]. Given the existing controversy around the influence of game location on physical and technical-tactical demands, it seems appropriate to analyse both in professional soccer players to help coaches design and structure their weekly routines properly, identifying the strong and weak indicators of the team, maintaining the workload of the strong indicators and increasing the workload of the weak indicators.

It seems that when attempting to clarify the interaction between physical, behavioural, technical and tactical variables of soccer performance considering game context [22], a holistic research may be needed. To the best of the authors’ knowledge, no research has focused on team success over an entire season considering playing positions and considering the analysis of technical-tactical and physical KPIs. Therefore, the aim of this study was to analyse the physical demands and technical-tactical actions for each playing position according to game location (i.e., playing at home vs. away) and final outcome (i.e., win or lose) in professional soccer players.

## Materials and methods

### Experimental design

The current investigation was descriptive and based on an observational methodology applied to the acquired data and was performed and reported according to the Strengthening the Reporting of Observational Studies in Epidemiology (STROBE) criteria (http://www.strobe-statement.org) (von Elm et al., 2007), which were obtained from global positioning system (GPS) devices (i.e., APEX pod) and Wyscout^®^, instrument validated [23], a multiple-camera tracking system so as to analyse the physical demands and the technical-tactical actions encountered by professional soccer players attending to contextual factors: location (play at home or away) and final outcome (win or lose) in which the same soccer team played a total of 30 official matches during the season 2017/2018. Also, the players were classified by their playing position. Two UEFA qualified coaches observed each of the games to verify that formation was consistent throughout the game [24]. Data for those players who did not play the entire match were excluded for further analysis.

### Participants

A convenience sample was obtained from twenty-one professional male soccer players (age: 25.10 ± 3.56 years; height: 180.25 ± 5.38 cm; body mass: 75.56 ± 6.40 kg; body mass index [BMI]: 23.15 ± 1.20 kg/m^2^), belonged to same soccer team of the Spanish Second Division (Table 1). All the players trained around 10 h per week and played an official match during the weekend (~ 5-6 conditioned sessions + 1 game per week). Subjects were assigned one playing position by the head coach. Playing positions were: central defenders (CD, n=5), wide defenders (WD, n=4), midfielders (MID, n=8) and forwards (F, n=4) [25][26].

**Table 1:** Participants

Position ^1^	n	Match	Age (years)	Height (cm)	Weight (kg)	BMI
CD	5	44	26.8 ± 3.49	187.6 ± 2.73	83.3 ± 2.68	23.39 ± 0.89
WD	4	44	24 ± 1.83	177.75 ± 5.68	70.52 ± 4.82	22.31 ± 0.70
MID	8	65	24.25 ± 3.85	177.75 ± 4.06	72.55 ± 6.24	22.94 ± 1.43
F	4	33	25.75 ± 4.19	180.25 ± 5.38	78.22 ± 1.97	24.10 ± 0.85

^1^CD: Central defender; WD: Wide defender; MID: Midfielder; F: Foward

Moreover, for further statistical analysis players were divided into four groups: (1) played at home and won (PHW), (2) played at home and lose (PHL), (3) play away and won (PAW) and (4) play away and lose (PAL), tied matches were eliminated. Goalkeepers were not included in the analysis due to their specific role during match-play [27]. Informed consent was obtained from all participants before the start of the study. Data was obtained from the daily monitoring of players, so that the professional club authorized researchers to use the data collection and no ethics committee was required [28, 29]. Otherwise, this study was conformed to the Declaration of Helsinki (2016) and was approved by a Local Ethics Committee of Universidad San Jorge, Spain, nº 08-20/21.

### Physical demands

Players’ physical demands were monitored during each match using a portable 18 Hz GPS unit and 600 Hz triaxial accelerometer (APEX pod accelerometer, MAPPS Technology and Bluetooth LE; STATSports; North Ireland). Randers et al. (2010) shown the validity, reproducibility and reliability of GPS devices. Each unit was introduced into an adjustable neoprene vest, inside a back pocket, positioned on the upper part of their backs, between the scapulaes. The physical demands selected for analysis were previously used in soccer players [30–32]: total distance (TD), moderate speed running distance (MSR; m >14.4 km·h^-1^), high-speed running distance (HSR; m >19.8. km·h^-1^), sprint distance (SPR; m >25.0 km·h^-1^), number of accelerations between 2 and 4 m·s^-2^ (Acc2-4) and above 4 m·s^-2^ (Acc>4), number of decelerations between 2 and 4 m·s^-2^ (Dec2-4) and above 4 m·s^-2^ (Dec>4). Post-match, data were downloaded and analysed using a customized software package (Apex, Statsports, Irlanda, Versión 1.2). Dwell time or minimum effort duration (MED) used in our variables were of 0,5 s in accelerations (Acc) and decelerations (Dec), and 1 s in sprint distance (SPR), high-speed running distance (HSR) and moderate speed running distance (MSR).

### Technical-tactical actions

Data related to technical-tactical actions were obtained from Wyscout^®^ (Chiavari, Italy), a computerized multiple-camera tracking system based on the OPTA^®^ (Spain) track analysis tool [1]. Stadiums’ cameras positioned at roof level captured players’ actions and were analysed using proprietary software [24]. The OPTA^®^ (Spain) reliability was identified with acceptable levels, showed an intra-class correlation coefficient varied from 0.88 to 1.00 [33]. Moreover, this technical-tactical Wyscout^®^ variables have been used and analysed in previous studies [1, 34]. For this investigation, were selected 19 variables, which were classified into three categories: (1) General indicators (GI), (2) defensive indicators (DI) and (3) offensive indicators (OI). (1) GI selected: Game volume (add DI and OI) (GV); (2) DI analysed: Defensive volume (add all of DI) (DV), nº interceptions (IN), nº opposing pitch interceptions (OPIN), clearances (CL), aerial duels (AD), aerial duels won (ADW); (3) OI used: Offensive volume (add all of OI) (OV), total pass (TP), total pass success (TPS), forward pass (FP), forward pass success (FPS), attack zone pass (AZP), turnover (TO), goal shot (GS), goal shot on target (GST), crosses (CR) crosses success (CRS) and dribbles (DR).

### Statistical analysis

Standard statistical methods were used for the calculation of the means and standard deviations (SD). Normal distribution and homogeneity of variances was examined by Shapiro-Wilk and Levene tests. The two-way ANOVA with the Tukey post hoc test was used to assess the impact of the interaction of both factors (i.e., match location and final outcome) on the external load responses encountered by soccer players. Statistical analyses were conducted using SPSS for Windows version 25.0 (SPSS Inc., Chicago, IL, USA). Statistical significance was set at p<0.05. Cohen’s effect size (ES) was used to evaluate practical differences between groups [35]. Thresholds for ES statistics were 0.2, trivial; 0.6, small; 1.2, moderate; 2.0, large; 2.0, very large; and >4.0, extremely large [36]. A threshold value of 0.2 between-subject standard deviations was set as the smallest worthwhile change (SWC), and unclear effect was then based on the disposition of the confidence interval for the mean difference to this smallest worthwhile effect.

## Results

The physical demands that soccer players face for each playing position and for all players when they play home or away and their team win or lose are compared and shown in Tables 2 and 3. For all players, higher TD (p = 0.045; ES = 0.24, S) was covered and greater Dec2-4 (p = 0.001; ES = 0.68, M) was performed when the team plays at home and lose. In addition, players performed more SPR when the team plays at home and wins (p = 0.009; ES = − 0.81, M) in comparison to the team playing at home and lose. Otherwise, MID (p = 0.012; ES = 0.79, M) and FW (p = 0.027; ES = 1.52, L) performed higher Dec2-4 when the team play at home and lose in comparison to the team playing at home and win. In addition, WD covered higher SPR (p = 0.026; ES = 1.01, L), when the team play at home and lose in comparison to when the team playing at home and win.

**Table 2:** Descriptive of the physical demands (mean ± SD) encountered by soccer players for each playing position and for all players when play at home or away, and their team win or lose

	CD				WD				MID			
	Home		Away		Home		Away		Home		Away	
	Win	Lose	Win	Lose	Win	Lose	Win	Lose	Win	Lose	Win	Lose
TD	10,311 ± 276	10,645 ± 713	10,262 ± 664	10,124 ± 480	10,820 ± 206	10,851 ± 997	10,784 ± 603	10,686 ± 498	11,987 ± 722	11,735 ± 607	11,647 ± 641	11,355 ± 861
MSR	1549 ± 262	1677 ± 397	1577 ± 285	1590 ± 351	2231 ± 237	2217 ± 840	2147 ± 288	2290 ± 315	2693 ± 5429	2555 ± 469	2560 ± 446	2559 ± 516
HSR	431 ± 97	402 ± 175	353 ± 83	387 ± 113	766 ± 123	742 ± 452	662 ± 123	770 ± 210	732 ± 272	596 ± 144	673 ± 214	620 ± 202
SPR	71 ± 35	53 ± 40	54 ± 11	73 ± 45	193 ± 87	189 ± 179	106 ± 44	174 ± 74	136 ± 85	62,97 ± 46,03	112 ± 68	94 ± 54
Acc2-4	151 ± 33	174 ± 63	136 ± 68	158 ± 29	173 ± 17	186 ± 24	144 ± 67	160 ± 36	166 ± 41	185 ± 25	163 ± 65	163 ± 38
Acc>4	11 ± 5	15 ± 10	13 ± 7	13 ± 8	19 ± 8	20 ± 5	20 ± 6	16 ± 10	12 ± 6	11 ± 4	16 ± 5	13 ± 7
Dec2-4	122 ± 30	162 ± 44	132 ± 58	136 ± 24	149 ± 12	162 ± 17	128 ± 55	151 ± 17	161 ± 34	190 ± 29	161 ± 59	162 ± 35
Dec>4	21 ± 9	22 ± 12	20 ± 8	22 ± 12	38 ± 8	37 ± 11	35 ± 13	30 ± 17	30 ± 14	28 ± 8	33 ± 10	28 ± 17

	FW				All			
	Home		Away		Home		Away	
	Win	Lose	Win	Lose	Win	Lose	Win	Lose
TD	10,827 ± 715	11,032 ± 320	11,049 ± 669	11,056 ± 733	10,992 ± 791	11,240 ± 909	11,009 ± 827	10,847 ± 824
MSR	2149 ± 437	2126 ± 190	2360 ± 238	2258 ± 309	2202 ± 577	2237 ± 548	2189 ± 514	2219 ± 550
HSR	808 ± 231	681 ± 142	850 ± 120	769 ± 283	685 ± 243	604 ± 241	620 ± 226	626 ± 246
SPR	233 ± 101	144 ± 72	266 ± 92	266 ± 201	152 ± 96	103 ± 96	122 ± 89	130 ± 105
Acc2-4	146 ± 24	170 ± 11	168 ± 30	150 ± 34	160 ± 32	178 ± 31	153 ± 61	160 ± 34
Acc>4	20 ± 8	22 ± 2	19 ± 10	15 ± 9	15 ± 8	15 ± 6	16 ± 7	14 ± 9
Dec2-4	125 ± 18	149 ± 10	136 ± 20	127 ± 27	142 ± 30	169 ± 32	142 ± 53	148 ± 30
Dec>4	27 ± 9	36 ± 11	25 ± 13	23 ± 13	30 ± 12	31 ± 10	29 ± 12	26 ± 15

CD: central defenders; WD: wide defenders; MID: Midfielders; FW: forwards; TD: total distance; MSR: distance covered above 14.4 km·h^−1^; HSR: distance covered above 19.8. km·h^−1^; SPR: distance covered above 25.0 km·h^−1^; Acc2-4: number of accelerations between 2-4 m·s^−2^; Acc>4: number of accelerations above 4 m·s^−2^; Dec2-4: number of decelerations between 2-4 m·s^−2^; Dec>4: number of decelerations above 4 m·s^−2^

**Table 3:** Mean differences (MD, %) and effect sizes (ES; ±CL) of the physical demands (mean ± SD) encountered by soccer players for each playing position and for all players when play at home or away, and their team win or lose

	CD				WD				MID			
	Home		Away		Home		Away		Home		Away	
	MD (%)	ES; ±CL	MD (%)	ES; ±CL	MD (%)	ES; ±CL	MD (%)	ES; ±CL	MD (%)	ES; ±CL	MD (%)	ES; ±CL
TD	3.1	0.43; ±1.19 S	− 1.3	− 0.21; ±0.72 S	0.0	0.00; ±1.21	− 0.9	− 0.16; ±0.74	2.2	0.37; ±0.66 S	2.2	0.37; ±0.66 S
MSR	7.5	0.27; ±1.15 S	0.0	0.00; ±0.67	− 6.4	− 0.15; ±1.20	6.5	0.46; ±0.71 S	− 4.7	− 0.23; ±0.66 S	− 4.7	− 0.23; ±0.66 S
HSR	− 11.3	− 0.25; ±1.17 S	8.2	0.31; ±0.67 S	− 18.8	− 0.27; ±1.21 S	14.0	0.55; ±0.67 S	− 14.2	− 0.40; ±0.61 S	− 14.2	− 0.40; ±0.61 S
SPR	− 33.8	− 0.40; ±1.15 S	12.2	0.21; ±0.59 S	− 38.2	− 0.34; ±1.20 S	63.1	1.01; ±1.01 M*	− 52.8	− 0.89; ±0.89 M	− 12.6	− 0.15; ±0.59
Acc 2-4	13.9	0.32; ±1.03 S	46.5	0.53; ±0.79 S	7.4	0.48; ±1.03 S	31.1	0.41; ±0.83 S	15.5	0.55; ±0.58 S	17.9	0.25; ±0.64 S
Acc >4	68.5	1.44; ±1.43 L	− 9.8	− 0.13; ±0.64	10.3	0.25; ±0.87 S	− 34.1	− 0.60; ±0.62 S	− 22.4	− 0.30; ±0.71 S	− 39.5	− 0.62; ±0.62 M
Dec 2-4	34.3	0.80; ±0.89 M	17.5	0.30; ±0.79 S	8.7	0.68; ±1.03 M	− 34.9	0.58; ±0.84 S	19.6	0.79; ±0.80 M*	13.0	0.23; ±0.64 S
Dec >4	8.3	0.08; ±0.93	− 6.3	− 0.09; ±0.64	− 6.5	− 0.16; ±1.16	− 31.8	− 0.50; ±0.63 S	18.6	0.21; ±0.57 S	− 27.8	− 0.43; ±0.52 S

	FW				All			
	Home		Away		Home		Away	
	MD (%)	ES; ±CL	MD (%)	ES; ±CL	MD (%)	ES; ±CL	MD (%)	ES; ±CL
TD	2.1	0.37; ±0.78 S	0.0	0.00; ±0.87	1.8	0.24; ±0.50 S*	− 3.2	− 0.43; ±0.47 S
MSR	0.8	0.04; ±0.78	− 4.7	− 0.38; ±0.86 S	− 2.8	− 0.11; ±0.51	− 6.4	− 0.26; ±0.44 S
HSR	− 13.0	− 0.44; ±0.86 S	− 13.2	− 0.54; ±0.83 S	− 16.4	− 0.42; ±0.51 S	− 12.5	− 0.32; ±0.44 S
SPR	− 34.9	− 0.59; ±0.99 S	− 26.2	− 0.61; ±0.84 M	− 49.9	− 0.81; ±0.81 M**	− 16.0	− 0.23; ±0.44 S
Acc 2-4	18.0	1.17; ±1.17 M	− 11.8	− 0.49; ±0.85 S	5.9	0.39; ±0.50 S	65.2	0.68; ±0.68 M
Acc >4	34.3	0.42; ±0.70 S	− 10.7	− 0.10; ±0.90	− 17.0	− 0.36; ±0.51 S	− 6.3	− 0.08; ±0.45
Dec 2-4	20.3	1.52; ±1.52 L*	− 8.1	− 0.38; ±0.84 S	12.2	0.68; ±0.69 M**	50.7	0.73; ±0.73 M
Dec >4	46.7	0.59; ±0.78 S	− 33.9	− 0.37; ±0.82 S	− 11.8	− 0.30; ±0.51 S	3.8	0.05; ±0.44

CD: central defenders; WD: wide defenders; MID: Midfielders; FW: forwards; TD: total distance; MSR: distance covered above 14.4 km·h^−1^; HSR: distance covered above 19.8. km·h^−1^; SPR: distance covered above 25.0 km·h^−1^; Acc2-4: number of accelerations between 2-4 m·s^−2^; Acc>4: number of accelerations above 4 m·s^−2^; Dec2-4: number of decelerations between 2-4 m·s^−2^; Dec>4: number of decelerations above 4 m·s^−2^

Standardized effect size thresholds: S. small; M. moderate; L. large; VL. very large; EL. extremely large

*Significant level set at p<0.05; ** Significant level set at p<0.01

The technical-tactical actions encountered by soccer players for each playing position and for all players when play at home or away and their team win or lose are compared and shown in Tables 4 and 5. For all players, playing at home and winning demanded higher DV (p =0.014; ES = − 1.49, L) and IN (p =0.031; ES = − 1.40, L) in comparison to playing at home and losing. Regarding playing position, MID performed greater GV, DV, IN and TPS (p = 0.011–0.043; ES = − 0.89–1.09, M) and FW obtained higher GV, DV and IN (p = 0.011–0.048; ES = − 0.64–0.89, M) when the team plays at home and wins compared to playing at home and losing. In addition, CD recorded higher GV and TPS (p = 0.044–0.047; ES = 0.17–0.88, S to M) when the team plays at home and loses in comparison to playing at home and winning. On the other side, FW registered higher FP and AZP (p = 0.028–0.029; ES = 0.99–1.16, M) when playing away and losing in comparison to playing away and winning. However, FW performed higher GST (p = 0.036; ES = − 1.21, L) when the team plays away and wins compared to playing away and losing.

**Table 4:** Descriptive of the technical-tactical actions (mean ± SD) encountered by soccer players for each playing position and for all players when play at home or away, and their team win or lose

	CD				WD				MID				FW				All			
	Home		Away		Home		Away		Home		Away		Home		Away		Home		Away	
	Win	Lose	Win	Lose	Win	Lose	Win	Lose	Win	Lose	Win	Lose	Win	Lose	Win	Lose	Win	Lose	Win	Lose
GV	56.7± 14.1	62.7± 16.5	51.6± 15.0	64.2± 14.8	60.3± 12.6	54.3± 15.7	65.4± 10.2	61.5± 14.8	68.7± 15.9	55.3± 12.5	59.5± 15.2	62.4± 15.9	33.9± 8.4	24.8± 8.2	31.4± 1.4	32.1 ± 9.6	569.1± 85.9	516.8± 98.1	537.2± 65.7	541.7 ±71.8
OV	41.2± 12.8	48.7± 15.8	37.5± 14.4	48.1± 15.5	50.3± 12.1	44.5± 14.3	51.3± 11.0	49.9± 13.4	55.0± 14.5	45.1± 12.5	45.7± 12.6	50.2± 15.1	27.7± 6.2	22.7± 8.7	25.3± 2.9	26.5 ± 6.6	457.3± 84.9	426.4± 99.5	414.3± 76.3	434 ±80.5
DV	15.5± 5.3	14.0± 2.9	14.1± 5.0	16.1± 4.5	10.0± 3.4	9.8± 3.0	14.1± 4.1	11.6± 4.3	13.7± 5.0	10.2± 4.4	13.8± 5.4	12.2± 3.9	6.2± 3.0	2.2± 2.0	6.1± 2.4	5.6± 3.7	111.8± 14.2	90.4± 11.8	122.8± 11.7	107.6 ±17.9
IN	6.0± 2.6	5.9± 2.6	4.6± 2.8	6.0± 2.6	4.4± 1.9	4.7± 2.0	7.2± 2.4	6.1± 2.9	9.1± 3.7	5.6± 2.4	8.1± 4.1	8.3± 3.0	2.9± 1.7	1.2± 1.2	3.3± 1.7	3.0± 2.4	59.1± 11.0	46.4± 4.1	62.7±7.6	58.2 ±10.1
OPIN	0.5± 0.7	0.7± 0.8	0.5± 0.6	0.6± 0.6	0.5± 0.9	0.3± 0.5	0.8± 1.1	0.9± 1.3	1.9± 1.4	1.8± 1.3	1.8 ± 1.9	2.3 ± 1.1	0.9± 1.4	0.7 ± 0.8	1.6 ± 0.9	1.0 ± 0.6	11.5 ± 4.2	11.2 ± 1.5	11.8 ± 3.7	13.1 ± 3.1
CL	6.0± 3.3	5.4 ± 2.7	6.7 ± 2.4	6.3 ± 3.0	2.8 ± 1.6	2.4 ± 1.3	4.1 ± 2.1	2.8 ± 2.1	2.2 ± 2.0	1.9 ± 2.9	2.4 ± 2.5	2.1 ± 1.7	1.2 ± 1.3	0.2 ± 0.4	1.6 ± 1.8	1.2 ± 0.9	28.9 ± 10.3	21.0 ± 10.3	36.3 ± 8.9	27.4 ± 8.0
AD	4.4 ± 2.3	4.1 ± 2.0	4.3 ± 2.9	4.2 ± 2.0	1.6 ± 1.8	1.9 ± 1.9	2.4 ± 1.9	2.3 ± 1.3	2.4 ± 1.6	1.7 ± 1.5	3.1 ± 2.2	2.3 ± 1.9	3.9 ± 2.7	3.0± 1.9	3.6 ± 2.9	3.5 ± 2.8	28.9 ± 8.6	25.4 ± 6.1	34.5 ± 13.9	29.1 ± 7.8
ADW	3.1 ± 2.3	2.9 ± 1.6	2.8 ± 2.1	2.6 ± 1.7	0.9 ± 1.3	0.9 ± 0.9	1.5 ± 1.7	1.0 ± 1.1	1.2 ± 1.5	0.9 ± 1.4	1.5 ± 2.1	1.3 ± 1.2	2.0 ± 1.7	0.5± 1.2	1.6± 1.3	1.6±1.7	15.6 ± 5.3	12.4 ± 3.5	18.3 ± 9.6	13.1 ± 3.8
TP	40.4 ± 12.3	48.0 ± 15.8	37.0 ± 14.4	47.0 ± 15.7	48.3 ± 11.8	42.9 ± 14.5	49.1 ± 10.5	48.1 ± 13.2	52.7 ± 14.7	42.7 ± 12.0	42.0 ± 12.8	48.7 ± 15.4	22.7± 6.6	18.5± 6.9	20.4± 2.4	22.9± 6.7	427.8± 82.1	396.8± 98.7	383.5± 76.5	409.8± 78.3
TPS	32.9± 12.5	41.2± 13.4	30.8± 9.2	35.0± 14.1	35.6 ± 9.8	34.0± 13.5	37.1± 8.3	37.2± 10.9	42.5± 12.6	32.8± 10.8	33.1± 10.4	38.9± 13.2	15.4± 5.6	13.2± 5.3	14.6± 3.2	16.0± 7.3	336.3± 84.4	310.0 ± 80.4	294.3 ± 61.6	319.7± 73.9
FP	30.8 ± 9.2	35.0 ± 14.1	28.2± 10.0	35.9± 12.0	30.2± 7.3	24.5± 7.1	30.4± 8.6	27.9± 8.7	33.1± 11.3	28.6± 9.3	26.1± 10.1	32.1± 11.9	10.2± 4.6	8.8± 5.2	8.0± 3.0	12.2± 3.9	265.3± 47.3	248.8± 67.2	242.2± 42.4	260.8± 47.1
FPS	23.3± 9.5	28.4± 11.9	20.5± 9.3	27.9 ± 12.1	18.8 ± 4.9	16.7 ± 5.4	19.1 ± 5.8	18.5 ± 6.3	24.7 ± 9.8	20.1 ± 7.5	17.8 ± 8.1	23.6± 10.3	4.8 ± 3.1	4.3 ± 3.3	4.1 ± 1.8	6.7 ± 3.3	187.4 ± 48.7	173.2 ± 51.2	160.5± 26.0	184.3± 43.4
TO	4.4 ± 2.9	4.6 ± 2.3	3.6 ± 2.0	4.3 ± 2.3	6.1 ± 3.0	7.5 ± 3.8	6.4 ± 1.9	6.7 ± 2.1	6.7 ± 3.3	6.1 ± 3.3	7.3 ± 3.9	6.9 ± 2.9	9.0 ± 2.6	5.8 ± 3.9	9.0 ± 3.6	9.0± 4.2	68.4± 8.9	67.6± 5.1	69.5± 9.0	71.1± 11.3
GS	0.3± 0.6	0.4± 0.5	0.3± 0.5	0.6± 0.8	0.3± 0.6	0.2± 0.7	0.0± 0.0	0.1± 0.3	1.1 ± 0.9	1.7± 1.3	1.2± 0.9	0.7 ± 0.9	3.0 ± 1.9	1.3± 1.6	3.0 ± 2.0	1.8 ± 1.3	12.8 ± 4.2	10.8 ± 4.8	10.3 ± 2.6	10.3 ± 2.9
GST	0.2 ± 0.4	0.1 ± 0.3	0.3 ± 0.5	0.5 ± 0.7	0.1 ± 0.3	0.0 ± 0.0	0.0 ± 0.0	0.1 ± 0.3	0.5 ± 0.6	0.9 ± 0.9	0.6 ± 0.6	0.4 ± 0.6	2.2 ± 1.8	0.8 ± 0.9	2.4 ± 1.4	1.0 ± 1.2	8.3 ± 2.5	5.8 ± 2.2	7.2 ± 1.5	6.0 ± 2.9
AZP	0.4 ± 0.7	0.7 ± 1.2	0.2 ± 0.4	0.9 ± 1.2	10.6 ± 5.2	11.5 ± 7.8	8.4 ± 5.0	10.1 ± 5.5	8.2 ± 4.8	8.3 ± 7.5	6.1 ± 4.9	6.3 ± 4.8	9.4 ± 2.9	8.2 ± 4.3	5.7 ± 2.9	9.4 ± 3.4	79.0 ± 22.2	77.6 ± 37.3	58.0± 17.8	74.4 ± 20.7
CR	0.1 ± 0.2	0.0 ± 0.0	0.0 ± 0.0	0.1 ± 0.3	2.0 ± 1.5	3.1 ± 2.8	1.3 ± 1.3	2.0 ± 1.8	1.9 ± 2.9	2.3 ± 2.5	1.2 ± 1.6	1.0 ± 1.3	0.3 ± 0.5	0.2 ± 0.4	0.3 ± 0.5	0.9 ± 1.0	11.1 ± 4.9	15.4 ± 8.2	8.2 ± 4.4	11.1 ± 4.5
CRS	0.0 ± 0.0	0.0 ± 0.0	0.0 ± 0.0	0.0± 0.0	0.2 ± 0.5	0.7 ± 0.7	0.5 ± 0.7	0.2 ± 0.4	0.5 ± 0.7	0.5± 0.7	0.5 ± 0.8	0.3 ± 0.6	0.0 ± 0.0	0.0 ± 0.0	0.0± 0.0	0.2 ± 0.3	2.7 ± 2.1	2.8 ± 1.5	3.0 ± 1.9	1.9 ± 0.8
DR	0.4 ± 0.9	0.4 ± 0.7	0.3 ± 0.5	0.5 ± 0.8	1.6 ± 1.7	1.4 ± 1.2	2.4 ± 1.7	1.7 ± 1.3	1.2 ± 1.7	0.7 ± 1.1	1.9 ± 2.1	0.8 ± 0.8	2.0 ± 1.3	2.8 ± 1.8	1.8 ± 1.7	1.7± 1.2	18.1 ± 6.8	18.8 ± 3.1	17.5 ± 2.7	14.0 ± 4.9

CD: central defenders; WD: wide defenders; MID: Midfielders; FW: forwards; GV: game volume; OV: offensive volume; DV: defensive volume; IN: interceptions; OPIN: opposing pitch interceptions; CL: clearances; AD: aerial duels; ARW: aerial duels won; TP: total pass; TPS: Total pass success; FP: forward. pass; FPS: forward pass success; TO: turnover; GS: goal shot; GST: goal shot on target; AZP: attack zone pass; CR: crosses; CRS: crosses success; DR: dribbles

**Table 5:** Mean differences (%) and effect sizes (ES; ±CL) of the technical-tactical actions encountered by soccer players for each playing position and for all players when play at home or away, and their team win or lose

	CD				WD				MID				FW				All			
	Home		Away		Home		Away		Home		Away		Home		Away		Home		Away	
	MD (%)	ES; ±CL	MD (%)	ES; ±CL	MD (%)	ES; ±CL	MD (%)	ES; ±CL	MD (%)	ES; ±CL	MD (%)	ES; ±CL	MD (%)	ES; ±CL	MD (%)	ES; ±CL	MD (%)	ES; ±CL	MD (%)	ES; ±CL
GV	10.2	0.32; ±0.74 S	26.0	0.88; ±0.88 M*	− 11.1	− 0.43; ±0.75 S	− 7.5	− 0.36; ±0.65 S	− 21.9	− 1.09; ±1.09 M*	5.3	0.19; ±0.56	− 29.0	− 0.89; ±0.91 M*	− 2.1	− 0.09; ±0.69	− 9.5	− 0.53; ±0.97 S	0.7	0.05; ±0.88
OV	17.0	0.40; ±0.74 S	30.1	0.77; ±0.77 M	− 12.7	− 0.45; ±0.76 S	− 4.0	− 0.16; ±0.66	− 19.9	− 0.83; ±0.83 M	9.3	0.29; ±0.56 S	− 22.8	− 0.60; ±0.94 M	− 2.1	0.10; ±0.72	− 7.3	− 0.33; ±0.97 S	4.7	0.22; ±0.89 S
DV	− 6.5	− 0.23; ±0.64 S	18.1	0.44; ±0.68 S	− 2.0	− 0.06; ±0.72	− 20.6	− 0.65; ±0.66 M	− 30.9	− 0.99; ±0.99 M*	− 7.9	− 0.20; ±0.57 S	− 42.1	− 0.81; ±0.89 M*	− 20.5	− 0.38; ±0.75 S	− 19.0	− 1.49; ±1.49 L*	− 13.2	− 0.96; ±0.96 M
IN	1.5	0.03; ±0.67	35.6	0.43; ±0.67 S	5.9	0.13; ±0.76	− 20.3	− 0.51; ±0.65 S	− 39.6	− 1.05; ±1.05 M*	− 2.1	− 0.04; ±0.57	− 35.5	− 0.64; ±0.95 M*	− 19.7	− 0.30; ±0.77 S	− 20.4	− 1.40; ±1.40 L*	− 7.8	− 0.53; ±0.85 S
OPIN	18.9	0.40; ±1.08 S	− 6.0	− 0.20; ±0.99 S	− 39.2	− 1.16; ±1.17 L	− 4.2	− 0.07; ±0.97	− 2.0	− 0.04; ±0.72	− 0.5	− 0.01; ±0.67	− 43.1	− 0.93; ±1.33 M	− 32.4	− 0.94; ±0.94 M	2.8	0.08; ±0.83	13.2	0.37; ±0.92 S
CL	0.9	0.02; ±0.63	− 13.3	-0.24; ±0.65 S	− 29.7	− 0.66; ±0.76 M	− 22.7	− 0.36; ±0.70 S	− 33.0	− 0.52; ±0.82 S	− 23.5	− 0.41; ±0.68 S	− 48.6	–	− 38.9	− 0.95; ±1.05 M	− 29.6	− 0.84; ±1.00 M	− 25.8	− 0.94; ±0.94 M
AD	− 12.0	− 0 0.21; ±0.77 S	7.1	0.11; ±0.68	25.1	0.42; ±0.90 S	24.0	0.33; ±0.70 S	0.5	0.01; ±0.73	− 13.2	− 0.25; ±0.62 S	− 29.4	− 0.43; ±0.87 S	− 7.2	− 0.10; ±0.89	− 11.2	− 0.41; ±0.94 S	− 13.3	− 0.42; ±0.92 S
ADW	− 6.0	− 0.09; ±0.73	2.5	0.04; ±0.71	0.8	0.01; ±0.99	− 31.3	− 0.57; ±0.87 S	21.4	0.24; ±1.06 S	− 24.4	− 0.44; ±0.81 S	51.1	–	− 3.1	− 0.05; ±0.92	− 19.2	− 0.58; ±0.94 S	− 24.3	− 0.67; ±0.94 M
TP	17.2	0.41; ±0.74 S	29.1	0.73; ±0.73 M	− 13-0	− 0.43; ±0.76 S	− 3.3	− 0.13; ±0.66	− 20.8	− 0.82; ±0.82 M	15.9	0.44; ±0.56 S	− 21.1	− 0.55; ±0.91 S	0.0	0.30; ±0.71 S	− 7.9	− 0.34; ±0.99 S	6.9	0.30; ±0.89 S
TPS	27.5	0.59; ±0.70 S	8.1	0.17; ±0.78*	− 7.9	− 0.22; ±0.77 S	− 1.5	− 0.05; ±0.66	− 24.5	− 0.89; ±0.89 M*	17.1	0.45; ±0.56 S	− 15.6	− 0.34; ±0.89 S	2.2	0.06; ±0.73	− 7.6	− 0.29; ±0.94 S	7.7	0.30; ±0.87 S
FP	8.1	0.17; ±0.78	28.0	0.73; ±0.73 M	− 19.6	− 0.72; ±0.72 M	− 8.6	− 0.31; ±0.67 S	− 16.5	− 0.53; ±0.66 S	21.8	0.48; ±0.56 S	− 20.8	− 0.34; ±0.91 S	54.7	0.99; ±0.99 M*	− 7.6	− 0.31; ±1.00 S	7.5	0.36; ±0.88 S
FPS	20.7	0.38; ±0.73 S	34.4	0.70; ±0.70 M	− 12.0	− 0.41; ±0.75 S	− 3.3	− 0.10; ±0.67	− 20.9	− 0.60; ±0.65 M	30.9	0.55; ±0.56 S	24.0	0.28; ±0.84 S	63.3	0.78; ±0.81 M	− 8.1	− 0.28; ±0.97 S	13.1	0.55; ±0.85 S
TO	2.7	0.04; ±0.73 S	36.4	0.48; ±0.68 S	21.6	0.32; ±0.74 S	1.5	0.04; ±0.66	− 15.6	− 0.30; ±0.67 S	− 9.4	− 0.17; ±0.56	− 43.0	− 1.01; ±1.01 M	− 1.3	− 0.02; ±0.80	− 0.7	− 0.06; ±0.87	1.9	0.13; ±0.87
GS	− 12.9	− 0.51; ±1.08 S	41.4	1.09; ±1.09 M	68.2	–	–	–	33.0	0.55; ±0.78 S	− 17.6	− 0.41; ±0.75 S	− 13.8	− 0.26; ±1.06 S	− 38.6	− 0.93; ±0.94 M	− 18.1	− 0.42; ±1.00 S	− 0.6	− 0.02; ±0.88
GST	0.0	–	26.0	0.77; ±0.99 M	–	–	–	–	24.8	0.52; ±0.96 S	3.9	0.14; ±0.91	28.4	− 0.53; ±0.99 S	− 44.9	− 1.21; ±1.21 L*	− 31-1	− 1.00; ±1.00 M	− 22.3	− 0.67; ±0.83 M
AZP	44.2	0.56; ±1.57 S	54.2	1.06; ±1.06 M	− 1.3	− 0.02; ±0.77	38.6	0.38; ±0.69 S	− 26.5	− 0.31; ±0.70 S	2.4	0.03; ±0.57	− 20.6	− 0.42; ±0.94 S	72.4	1.16; ±1.17 L*	− 6.2	− 0.15; ±1.00	29.8	0.79; ±0.91 M
CR	–	–	–	–	12.8	0.16; ±0.80	21.8	0.37; ±0.80 S	31.3	0.30; ±0.82 S	− 19.7	− 0.36; ±0.81 S	0.0	–	42.6	0.94; ±0.95 M	34.1	0.45; ±0.97 S	37.4	0.69; ±0.89 M
CRS	–	–	–	–	− 18.8	− 0.16; ±1.78	− 12.9	− 0.51; ±1.08 S	0.0	0.00; ±1.08	− 24.2	− 0.72; ±1.08 M	–	–	–	–	19.8	0.24; ±0.91 S	− 49.6	− 1.66; ±1.67 L
DR	− 29.3	–	68.2	1.54; ±1.54 L	− 13.3	− 0.23; ±0.84 S	− 9.6	− 0.17; ±0.73	− 5.8	− 0.09; ±0.95	− 35.7	− 0.81; ±0.82 M	0.7	0.01; ±0.93	8.4	− 0.12; ±0.92	10.8	0.29; ±0.83 S	− 23.4	− 0.91; ±0.91 L

CD: central defenders; WD: wide defenders; MID: Midfielders; FW: forwards; GV: game volume; OV: offensive volume; DV: defensive volume; IN: interceptions; OPIN: opposing pitch interceptions; CL: clearances; AD: aerial duels; ARW: aerial duels won; TP: total pass; TPS: Total pass success; FP: forward pass; FPS: forward pass success; TO: turnover; GS: goal shot; GST: goal shot on target; AZP: attack zone pass; CR: crosses; CRS: crosses success; DR: dribbles

Standardized effect size thresholds: S. small; M. moderate; L. large; VL. very large; EL. extremely large

*Significant level set at p<0.05

## Discussion

The aim of this study was to analyse the physical demands and technical-tactical actions for each playing position according to game location (i.e., local vs. visitor) and final outcome (i.e., win vs. lose) in elite professional players. The current study is ground-breaking due to the attempt to determine success in Spanish professional soccer games including physical and technical-tactical KPIs for each playing position in different context game to achieve a holistic approach. The main findings were as follows: (i) physical demands and the technical-tactical actions vary when two contextual game factors (i.e., game location and final outcome) are considered, (ii) higher TD covered and Dec 2-4 performed could be related when the team plays at home and loses for all players, MID and FW, (iii) greater number of SPR are exhibited by players when the team plays at home and wins, (iv) greater GV, DV and IN is recorded when the team plays at home and win for all players, MID and FW, (v) higher GV and TPS are performed by CD when the team plays at home and losing, and (vi) greater FP and AZP are recorded by players when the team plays away and loses.

There exists lack of scientific evidence regarding the KPIs influencing the final outcome of a game considering its location (i.e., home vs. away) since most of the studies assess team success across the seasons [12–15]. From a general overview, the analysis of physical KPIs showed that when a team played at home and won, their players covered greater SPR, whereas when the team was defeated, greater TD and Dec2-4 were identified. It is well known that sprints are the most repeated actions in goal situations [37]. This allows to suggest that greater SPR might be associated with success when playing at home as a high number of goal attempts would be created and, thus, greater probability to achieve victory would be increased. Despite both studies consider the same competitive standards, such discrepancies might be explained in relation with temporal factors given that there is a span of period of 11 seasons between the aforementioned study and the current work. It is well known that today soccer is more physically demanding than before and, therefore, a higher number of sprints in each game is shown [38, 39].

As score changes, the team which is behind needs to do greater physical efforts to reduce that difference and overcome the other [21]. This statement is supported by our findings since greater TD and Dec2-4 values were found as the home team loses. On the contrary, two previous studies reported increased values for total distance covered, and low (11-14 km/h) and moderate intensity running (14-19 km.h^-1^) on the side of the home team when they achieved victory in Brazilian third division championship [41]. However, it is widely accepted that low-intensity activities are not crucial in professional soccer performance [14]. A greater physical effort (i.e., higher total distance covered) does not guarantee success [42] as, shown in the current work, it might be associated with other cognitive, emotional or tactical factors than a lower physical performance [39]. Moreover, no significant differences were observed between won or lost matches when teams play away. In that context, it seems that KPIs such as technical-tactical efficacy might have a greater influence on success than physical KPIs [12, 22].

On the one hand, one of the most robust findings derived from time motion analysis are the different physical demands regarding playing positions [43]. Activity profiles and tactical demands are considered positional dependent in soccer, therefore, the analysis based on playing positions might be useful to know whether physical performance influences success. Analysis of playing positional data on physical performance showed differences between MID and FW as the game was played at home and when the team lost in Dec2-4. Such differences might be explained due to the fact that MID need to escape from an opponent and find a free space in order to receive a pass resulting in technical-tactical demands which implied an increased number of changes of direction. Accordingly, deaccelerations are essential within these changes of direction not only when attacking, but also as the team defends [44]. A higher number of Dec2-4 identified as the team loses at home might be an indicator of the presence of more changes of direction carried out associated to the difficulty in taking the ball away from the opponent and, thus, associated to less success. Moreover, it seems that a greater number of curvilinear runs has been observed for MID before taking possession of the ball [43], resulting in a higher number of Dec2-4. This can negatively contribute to take the ball from the opponent as MIDs need to brake making this issue especially hard. It is worth highlighting that ball possession has been identified as an indicator of success in soccer [18]. Similarly, an increased amount of Dec2-4 has been shown for FW when the team plays at home and loses. However, most of the studies reported that distance covered sprinting during attacks in FW seems to be essential in order to succeed [45]. These actions facilitate the attack actions requiring slips [46] to look for positive situations or to break into the opponent box [43].

On the other hand, WD show a high number of SPR when playing at home and losing the game in comparison with playing away and winning [45]. This might be attributed to the repeated efforts derived from attack-defend transitions needed to recover defensive positions [45]. These findings seem to be supported by ours as an acceleration is needed prior to decelerate [44]. Finally, no physical demand when analysing by playing position showed differences between playing at home or away when team wins. These findings may suggest that a greater physical effort could have no relation with achieving victory. Although a proper physical capacity may be favourable in order to deal with soccer conditional demands, there exist concerns regarding the connection between physical performance and competitive success [22]. Given this existing controversy, it seems noteworthy the clarification of the degree of technical-tactical participation associated with playing positions as it has been considered as an indicator of soccer performance to determine its influence on success [13, 47]. In these regards, the present study observed more significant GV, DV and IN when the team won playing at home.

A previous study concluded that defensive actions were more related with the accumulation of points in evenly-matched championships (i.e., Spanish soccer second league) [48]. Accordingly, those teams carrying out more interceptions, tackles [49] and winning aerial duels [50] were more likely to win the game. In addition, it seems that better teams are more efficient when applying defensive pressure near the opponent box [49] leading the opponent team to make mistakes and hindering their progression. This contributes to a greater amount of DV and IN to win games as home team. Of note, these differences between DV and IN are not found when team plays away as the contextual factor influences these types of actions when a team wins at home.

The analysis in relationship with technical-tactical actions across playing position showed some differences in CD, MID and FW. Playing at home and victory demanded higher values of GV, DV, IN and TPS for MID; GV, DV and IN for FW. Likewise, a previous study reported that when playing home, UEFA Champions League teams recovered the ball more frequently than when playing away [49]. The atmosphere when playing at home, having all the fans encouraging the players, is associated with increased aggressiveness and intentionality in players causing successful defensive actions [51]. Moreover, recovering the possession of the ball close to the opponent box has been identified as an influencing factor on success in soccer [7, 49], supporting, therefore, the findings reported in the current work for MID and FW in GV, DV and IN. Likewise, our study also Support previous findings [52] where a higher number of passes by MID contributed to increased chances to score a goal. Although passes accuracy is related to ball possession [53], this might occurred near the opponent box in order to be effective and contribute to win the game [19]. Regarding this, the MID might be decisive to succeed when playing at home.

Then, our findings showed that winning playing at home required more GST for FW, whereas losing playing away is more related to greater GV and TPS for CD, and AZP and FP for FW. Hence, a greater number of GV and TPS was associated to lose a game away in CD. In fact, ball possession far from the opponent box and with no intention to make a progress resulted ineffective [9]. Therefore, the accumulation of a greater number of GV and TPS in CD not only seems to influence victory, but also it is associated with losing games away. Likewise, a high number of attempts ot goal has been identified as a key KPI to win games [54] as well as to succeed at the end of a championship [1, 4, 51, 54–56]. The findings shown in the current work support the aforementioned discussed studies, but it determines a very novel aspect as FW is the only position that establishes GST as KPI to win games away. This may suggest that key role of FW should be to score goals and to focus on doing GST instead of trying to participate excessively in game creation as it has been observed that a great number of AZP and FP carried out by FW in a game are associated to lose games away.

Despite the findings shown in the present study, there are some limitations to be considered. First, either score dynamics or games ended in a tie were not considered. Then, tactics and game model used in each game were also not considered, remaining unknown physical demands under different models and strategies. Second, the wide midfielders have not been described because they were substituted in practically all the matches, which is why they were excluded from the present study since the sample was not significant. The third limitation comes from the sample size of the players who participated in the study. It would be interesting to have access to a greater number of players in order to obtain more representative results.

*Practical applications*.

The findings here provided might help sports practitioners understand that greater physical expenditure (i.e., greater amount of distance covered in different speed zones) during games seems to have no relationship with achieving victory. Therefore, it seems that fitness development should be aimed at dealing with the game physical demands derived from the coach’s proposal and minimising injury risk. Additionally, context variables are strongly influenced by playing position and not by final score [43], thus, the adoption of a position-specific approach for player conditioning would be potentially needed.

The main practical approach for coaches is the knowledge of the implications of the technical-tactical KPIs to win games. This could determine the strategical behaviour of the team and guide a successful model of play. During practices, coaches and practitioners would put special emphasis on that technical-tactical KPIs that have shown to be essential to achieve victory based on the context of the next game (i.e., home or away). This would help select players and thus enhance team performance linked to the individual characteristics. Therefore, further research is needed to clarify which physical demands and technical-tactical KPIs are key in defend-attack and attack-defend transitions given their outstanding importance in soccer games.

## Conclusions

The findings here reported support that physical demands and technical-tactical actions vary when contextual game factors (i.e., match location and final outcome) are considered. As such, higher TD covered and Dec 2-4 performed could be related when the team plays at home and loses for all players, MID and FW. In addition, greater number of SPR are exhibited when the team plays at home and wins. Moreover, greater GV, DV and IN are recorded when the team plays at home and win for all players, MID and FW. Otherwise, higher GV and TPS are performed by CD when the team plays at home and loses. Finally, greater FP and AZP are recorded when the team plays away and loses. Overall, greater physical performance was not associated with winning soccer games, therefore, the recognition of the implications of technical-tactical KPIs to win could improve the selection of training goals, model play and selection of players to achieve optimal team performance that could help win games.

## Data Availability

The datasets generated and analysed during the current study are not publicly available due to ethical restrictions, however, they are available from the corresponding author on reasonable request.

## Abbreviations

CD: Central defenders
WD: wide defenders
MID: midfielders
F: forwards
PHW: played at home and won
PHL: played at home and lose
PAW: play away and won
PAL: play away and lose
KPIs: key performance indicators
GPS: global positioning system
BMI: body mass index
TD: total distance
MSR: moderate speed running distance
HSR: high-speed running distance
SPR: sprint distance
Acc2-4: number of accelerations between 2-4 m·s^-2^
Acc>4: above 4 m·s^-2^
Dec2-4: above 4 m·s^-2^
MED: minimum effort duration
Acc: accelerations
Dec: decelerations
SPR: 1 s in sprint distance
HSR: high-speed running distance
MSR: moderate speed running distance
GI: General indicators
DI: defensive indicators
OI: offensive indicators
GV: Game volume
DV: Defensive volume
IN: no interceptions
OPIN: no opposing pitch interceptions
CL: clearances
AD: aerial duels
ADW: aerial duels won
OV: offensive volume
TP: total pass
TPS: total pass success
FP: forward pass
FPS: forward pass success
AZP: attack zone pass
TO: turnover
GS: goal shot
GST: goal shot on target
CR: crosses
CRS: crosses success
DR: dribbles
SD: standard deviations
ES: effect size
SWC: smallest worthwhile change
